# Quantitative EEG of frontal and central cortices during calm memory retrieval in schizophrenia

**DOI:** 10.1007/s44192-026-00565-7

**Published:** 2026-08-01

**Authors:** S. Khemthong, M. Rueankam, W. Chatthong

**Affiliations:** Division of Occupational Therapy, Faculty of Physical Therapy, Mahidol University, Nakhon Pathom, Thailand

**Keywords:** Theta relative power, QEEG, Schizophrenia, Working memory, Calmness, Brain mapping, Executive function

## Abstract

**Background:**

Altered neural oscillations are a hallmark of cognitive and emotional dysregulation in schizophrenia. Theta-band activity, particularly in frontal and central regions, has been associated with working memory, internal attention, and relaxed cognitive states. However, the role of theta dynamics during tasks that integrate memory retrieval with a calm, internally directed state remains unclear.

**Methods:**

Thirty individuals with schizophrenia participated in a structured memory recall task embedded within the Brain Mapping Performance (BMP) protocol. Three conditions were examined: calm (eyes closed with guided internal focus), resting (eyes open), and active memory task. Quantitative EEG (QEEG) was recorded from 12 scalp sites, with analyses focused on theta relative power and theta/beta ratio at frontal, central, and temporal sites (Fp1, Fz, F4, F7, Cz, T3). Behavioral performance and self-reported calmness were also assessed. Repeated-measures ANOVA was used to evaluate condition and electrode-site effects.

**Results:**

Theta relative power was highest during the calm condition and decreased during resting and memory task conditions (p < .001), with prominent activity at frontal-midline (Fz) and central (Cz) sites. The memory task was associated with reduced theta dominance and increased task demands, reflected by lower accuracy and longer completion time (p < .05). Theta/beta ratio followed a similar descriptive pattern, with higher values during calm states and lower values during active task engagement. Exploratory correlation analyses indicated associations between EEG activity and behavioral performance.

**Conclusion:**

Frontal and central theta activity may reflect modulation between internally directed calm states and active processing in schizophrenia. The highest theta activity observed during calm conditions suggests that internally focused states may support neural readiness, while task-related reductions reflect increased executive demand. These findings support the use of QEEG-based measures within occupation-integrated paradigms, such as BMP, for assessing and guiding cognitive-emotional regulation in psychiatric rehabilitation.

## Introduction

Schizophrenia is a chronic psychiatric disorder characterized by disruptions in cognition, emotion, and behavior, with impairments in working memory, attention, and executive function being among the most disabling features [17]. These deficits significantly affect daily functioning and long-term outcomes [11, 24]. Neurophysiological approaches, particularly quantitative electroencephalography (QEEG), provide valuable insights into these impairments by capturing dynamic patterns of brain oscillations associated with cognitive and affective processes [20, 22, 28].

Neural oscillations in the theta band (4–7 Hz) are widely associated with working memory, cognitive control, and internally directed attention. Frontal-midline theta activity, in particular, has been linked to top-down executive regulation and calm, focused mental states [6, 25]. In addition, frontal alpha activity has also been implicated in relaxed and internally oriented states, suggesting that multiple oscillatory mechanisms may support cognitive-emotional regulation under low external demand conditions [8, 12]. In schizophrenia, altered theta dynamics—often reflecting disrupted or compensatory neural activity—have been observed during memory and executive task demands [2, 16, 27].

Despite increasing interest in oscillatory biomarkers, few studies have examined how neural activity is modulated across cognitive states that integrate both memory processing and internally directed calmness. This distinction is clinically relevant, as individuals with schizophrenia often struggle to regulate internal attention while engaging in goal-directed tasks. Understanding how neural systems transition between internally focused calm states and active cognitive engagement may provide insights into mechanisms underlying executive dysfunction and emotional dysregulation. Previous studies have shown that working memory performance in schizophrenia is associated with altered frontal theta activity and executive dysfunction, supporting the relevance of examining memory-related neural dynamics under calm, internally focused conditions [3, 10, 18, 23].

In this study, we define “calmness” as an experimentally induced state characterized by eyes-closed conditions combined with guided internal attention and reduced sensory input. This operationalization distinguishes calmness from resting state (eyes open), which involves passive environmental monitoring, and from active task engagement, which requires sustained external attention and working memory processing. This distinction allows for a more precise investigation of how internally directed states influence cognitive performance [20, 25]. Although this calm condition shares some characteristics with conventional eyes-closed resting-state EEG, the inclusion of guided internal attention and intentional memory preparation distinguishes it from passive resting-state recording.

From a neuro-occupational perspective, executive functions —including working memory, attentional initiation, and inhibitory control—are fundamental for goal-directed behavior and participation in daily activities [9, 13, 26].

In schizophrenia, impairments in these domains interfere with the ability to initiate, sustain, and complete meaningful occupations. Occupational therapy frameworks emphasize the integration of cognitive, emotional, and environmental factors, suggesting that structured tasks designed to support calm and focused attention may facilitate functional engagement [4, 5, 14].

To investigate these processes, we employed the Brain Mapping Performance (BMP) protocol, an occupation-integrated QEEG assessment, to examine theta dynamics during a calm memory task in individuals with schizophrenia [4, 5]. The objectives of this study were: (1) to identify changes in theta relative power across calm, resting, and memory task conditions, and (2) to examine the relationships between theta activity and behavioral performance. We hypothesized that theta activity would be highest during internally directed calm states and would be modulated during active task engagement, particularly in frontal and central regions associated with executive function.

## Methods

### Participants

Thirty individuals (mean age = 38.9 ± 7.15 years; 10 male, 20 female; all right-handed) diagnosed with paranoid schizophrenia participated in this study. The sample size was determined based on prior QEEG studies using similar experimental designs and analytical approaches, including those conducted in schizophrenia populations, which have demonstrated adequate sensitivity for detecting within-subject effects in repeated-measures analyses [4, 5, 15]. All participants were receiving outpatient psychiatric care and met DSM-5 and ICD-10 diagnostic criteria for schizophrenia (F20.0). All participants were assessed using the Thai version of the Positive and Negative Syndrome Scale (PANSS) prior to EEG recording to ensure clinical stability. Individuals who exhibited acute psychotic symptoms or high positive symptom scores were excluded from the session. The PANSS Thai version has suggested acceptable criterion validity and interrater reliability in local populations [21]. Exclusion criteria included comorbid neurological conditions and current substance use disorders. All participants provided written informed consent prior to participation. This study did not include a healthy control group, as the primary aim was to examine within-group neural dynamics in individuals with schizophrenia within an occupational therapy framework.

### Ethical approval

All procedures performed in this study involving human participants were approved by the Central Institutional Review Board of Mahidol University (MU-CIRB Project ID: 2020/114.1505; COA No. 2020/103.0708), and by the Ethics Committee on Mental Health Research in Human Subjects, Department of Mental Health, Ministry of Public Health, Thailand (DMH.IRB Project ID: 031/2563, Version 2; COA No. 041/2563).

### Procedure and task design

**Fig. 1:**
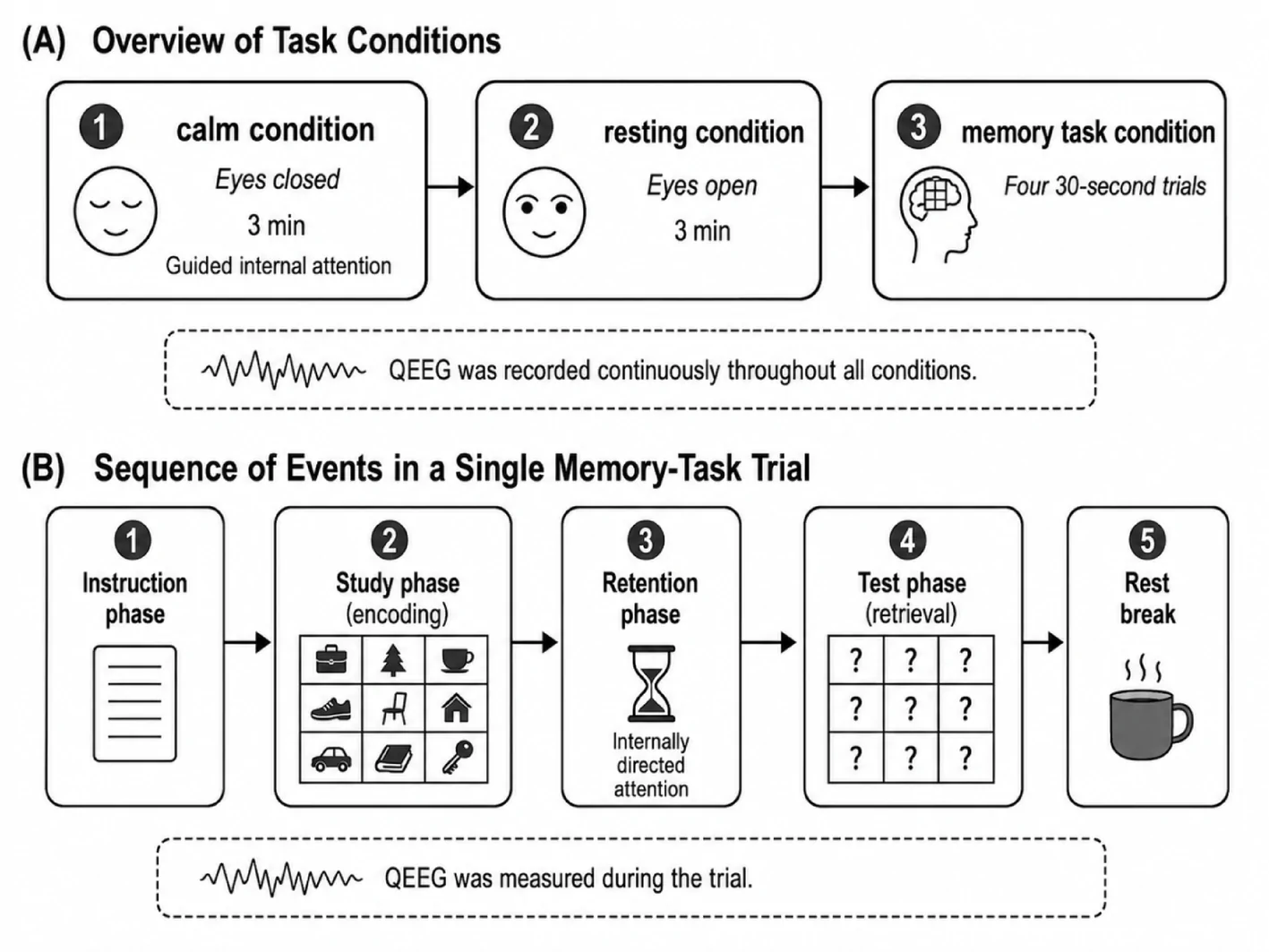
Experimental protocol and task schematic. **A** Overview of the three experimental conditions in sequential order: (1) calm condition (eyes closed with guided internal attention), (2) resting condition (eyes open), and (3) memory task condition. **B** Schematic representation of a single memory-task trial, illustrating the instruction, study (encoding), retention, and test (retrieval) phases, followed by a rest break

Participants completed a spatial memory recall task involving nine occupations, randomly arranged in a 3 × 3 matrix, based on the Thai Physical Activity Guideline. The task was delivered via a mobile application developed by the authors [1] (Fig. 1). Three cognitive conditions were assessed: (1) a 3-min calm condition with eyes closed, (2) a 3-min resting condition with eyes open, and (3) a memory task consisting of four 30-s trials.

During the memory task, participants were instructed to recall the nine occupations and their spatial locations within the matrix by touching the corresponding positions on the screen while maintaining a relaxed posture. The calm condition was designed to promote internally directed attention and intentional memory preparation, whereas the resting condition involved passive eyes-open monitoring without active recall demands.

QEEG was continuously recorded throughout all conditions using the BrainMaster Discovery 24E system within the Brain Mapping Performance (BMP) protocol.

### QEEG recording and analysis

QEEG data were recorded using a 12-channel system (BrainMaster Discovery 24E) based on the International 10–20 electrode placement system. Analyses focused on six electrode sites (Fp1, Fz, F4, F7, Cz, and T3), which have been consistently identified in prior QEEG-based occupational therapy research [4, 5] as key regions associated with executive function, attentional regulation, sensorimotor integration, and language-related processing.

EEG signals were recorded at a sampling rate of 250 Hz and bandpass filtered between.

1 and 30 Hz. Data were referenced to linked earlobes (A1–A2). Artifacts related to eye movements and muscle activity were removed using a combination of automated amplitude thresholding and manual visual inspection, following established preprocessing procedures [7, 20]. Automated thresholding was performed using an amplitude criterion exceeding ± 100 µV, followed by manual visual inspection to confirm artifact rejection. EEG data were processed using NeuroGuide Deluxe QEEG software (version 3.0.7.3; Applied Neuroscience, Largo, FL) and analyzed offline.

Continuous EEG data were segmented into non-overlapping 2-s epochs corresponding to each experimental condition (calm, rest, and memory task), and artifact-free segments were retained for analysis. Theta-band power was defined as spectral activity within the 4–7 Hz frequency range, while beta-band power was defined within the 13–30 Hz frequency range. Power spectral density was calculated for each frequency band using Welch’s method with 2-s epochs and 50% overlap. Relative theta power was calculated as the proportion of theta-band power relative to total spectral power across the 1–30 Hz range. Beta-band power used for exploratory correlation analyses was calculated using the same normalization procedure. No logarithmic transformation was applied prior to spectral estimation, and data were visually inspected for baseline stability before analysis.

The theta/beta ratio (4–7 Hz / 13–30 Hz) was computed as an index of attentional regulation and is presented for descriptive purposes only. This metric should be interpreted cautiously, as it may reflect multiple cognitive and affective processes depending on task context. Individual alpha peak frequency (IAF) was not controlled for in the present analysis, which may influence spectral band boundaries and contribute to inter-individual variability. Overall, this approach follows established QEEG methodologies and provides a reliable representation of theta activity across cognitive conditions [7, 20].

Statistical analyses were conducted using repeated-measures analysis of variance (ANOVA) to examine the effects of condition and electrode site. Where assumptions of sphericity were violated, Greenhouse–Geisser corrections were applied. Post hoc comparisons were performed using Bonferroni correction, and effect sizes were reported as partial eta squared (η^2^_P_). Exploratory Pearson correlation analyses were conducted to examine relationships between EEG measures and behavioral performance. No correction for multiple comparisons was applied; therefore, findings should be interpreted cautiously.

## Results

### Theta relative power across conditions and electrode sites

A repeated-measures ANOVA was conducted to examine the effects of condition (calm, rest, memory) and electrode site (Fp1, Fz, F4, F7, Cz, T3) on theta relative power. Because assumptions of sphericity were violated for repeated-measures factors, Greenhouse–Geisser corrections were applied where appropriate; the fractional degrees of freedom reported below reflect these corrected values. There was a significant interaction between condition and electrode site, F(6.643, 146.146) = 5.220, *p* < 0.001, η^2^_P_ = 0.19, indicating that changes in theta relative power varied across electrode locations depending on the condition.

There was also a significant main effect of condition, F(1.648, 36.258) = 4.264, *p* = 0.028, η^2^_P_ = 0.16, demonstrating that theta relative power differed significantly across calm, rest, and memory conditions. In addition, a significant main effect of electrode site was observed, F(1.868, 41.100) = 31.271, *p* < 0.001, η^2^_P_ = 0.59, indicating variability in theta power across different scalp regions.

Descriptive statistics (Table 1) showed that theta relative power was highest during the calm (eyes-closed) condition across all electrode sites, particularly at Fz (23.87 ± 10.88) and Cz (22.52 ± 11.76). The resting (eyes-open) condition showed intermediate values, while the memory condition suggested relatively lower theta values across most sites.

**Table 1:** Mean ± SD of theta relative power (%) at Each QEEG site across conditions

QEEG site	Calm (Eyes Closed)	Rest (Eyes Open)	Memory task
Fp1	20.50 ± 9.15	16.51 ± 4.97	18.21 ± 5.70
F7	19.61 ± 8.42	18.45 ± 5.04	17.83 ± 4.05
F4	21.97 ± 10.23	20.75 ± 5.47	20.12 ± 4.99
Fz	23.87 ± 10.88	22.99 ± 6.42	22.77 ± 6.04
Cz	22.52 ± 11.76	21.95 ± 6.20	20.85 ± 5.51
T3	17.37 ± 9.50	16.29 ± 6.83	14.40 ± 4.72

**Fig. 2:**
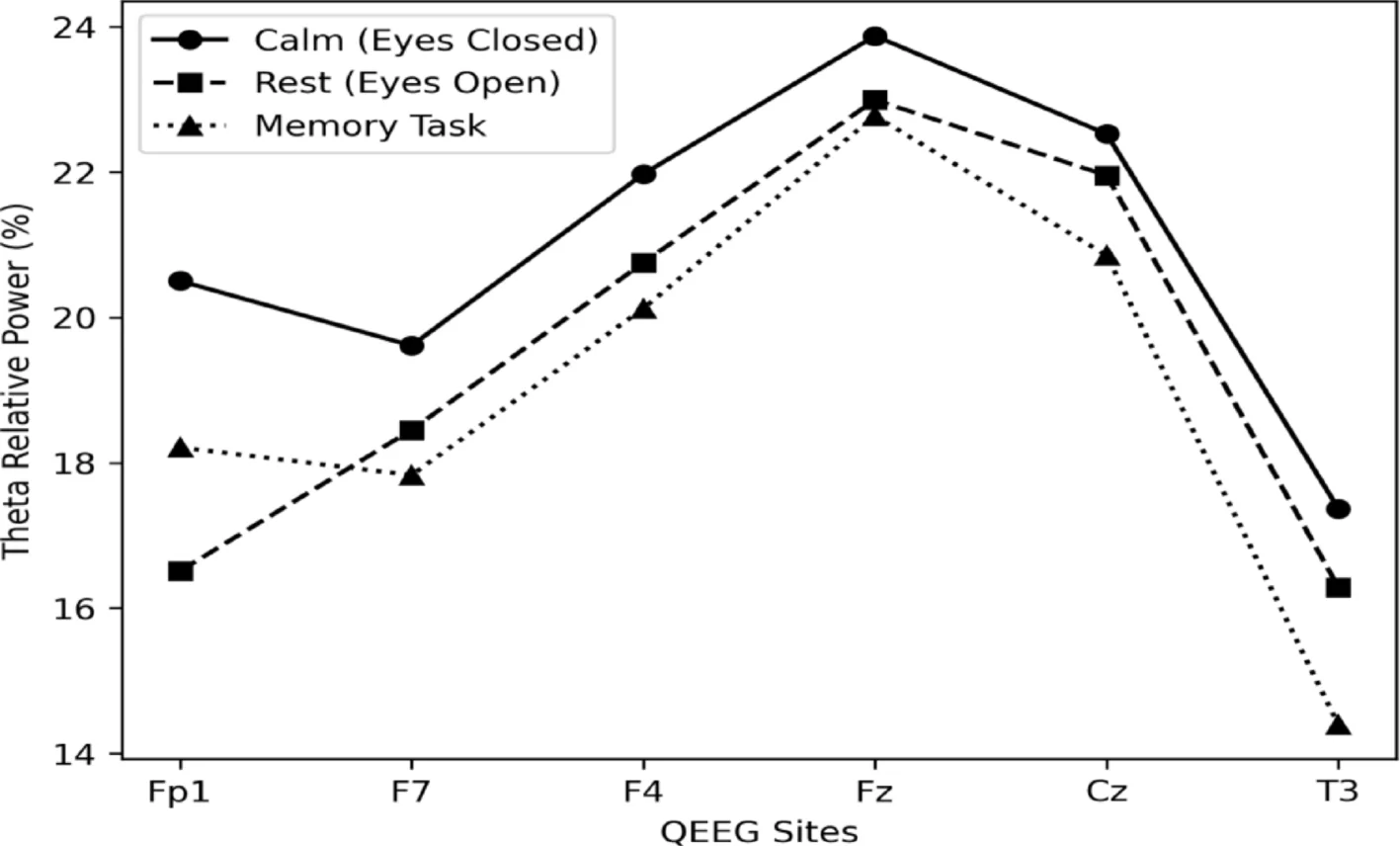
Theta relative power across calm, rest, and memory task conditions at six QEEG electrode sites. Solid, dashed, and dotted lines distinguish conditions in grayscale

Across electrode sites (Fig. 2), frontal and central regions (Fp1, Fz, Cz) consistently exhibited higher theta relative power compared with the temporal site (T3). Bonferroni-corrected post hoc comparisons showed significantly higher theta relative power during the calm condition compared with both the resting condition (*p* = 0.012) and memory task condition (p = 0.004), whereas no significant difference was observed between resting and memory task conditions (*p* > 0.05).

### Theta/beta ratio across conditions

Theta/beta ratios across the six QEEG sites under calm, rest, and memory conditions are presented in Table 2. This measure was included as a complementary descriptive index of attentional regulation across cognitive states, consistent with prior QEEG literature.

**Table 2:** Mean ± SD of theta/beta ratio at each QEEG site across conditions

QEEG site	Calm (Eyes Closed)	Rest (Eyes Open)	Memory task
Fp1	1.54 ± 1.02	1.18 ± 0.48	0.58 ± 0.38
F7	1.34 ± 0.87	1.03 ± 0.39	0.78 ± 0.27
F4	1.33 ± 0.92	1.08 ± 0.45	0.86 ± 0.43
Fz	1.56 ± 1.09	1.31 ± 0.63	1.24 ± 0.52
Cz	1.40 ± 1.18	1.19 ± 0.68	1.19 ± 0.51
T3	1.04 ± 1.12	0.76 ± 0.47	0.55 ± 0.33

Descriptive analysis showed that theta/beta ratios were highest during the calm condition, with peak values at Fz (1.56 ± 1.09) and Fp1 (1.54 ± 1.02). In contrast, lower ratios were observed during the memory condition, particularly at T3 (0.55 ± 0.33) and Fp1 (0.58 ± 0.38). The resting condition showed intermediate values across most electrode sites.

Overall, theta/beta ratios showed a decreasing descriptive trend from calm to rest to memory conditions across the majority of electrode sites. No inferential statistical analyses were conducted for theta/beta ratio; therefore, these findings are interpreted cautiously and used only to support the primary theta power results.

### Correlation between EEG activity and behavioral performance

Exploratory correlation analyses revealed significant relationships between EEG activity and behavioral performance measures. Although beta activity was not examined as a primary outcome in the repeated-measures ANOVA, it was included in the exploratory correlation analyses because beta oscillations are relevant to attentional control, executive demand, and task-related cognitive engagement.

**Fig. 3:**
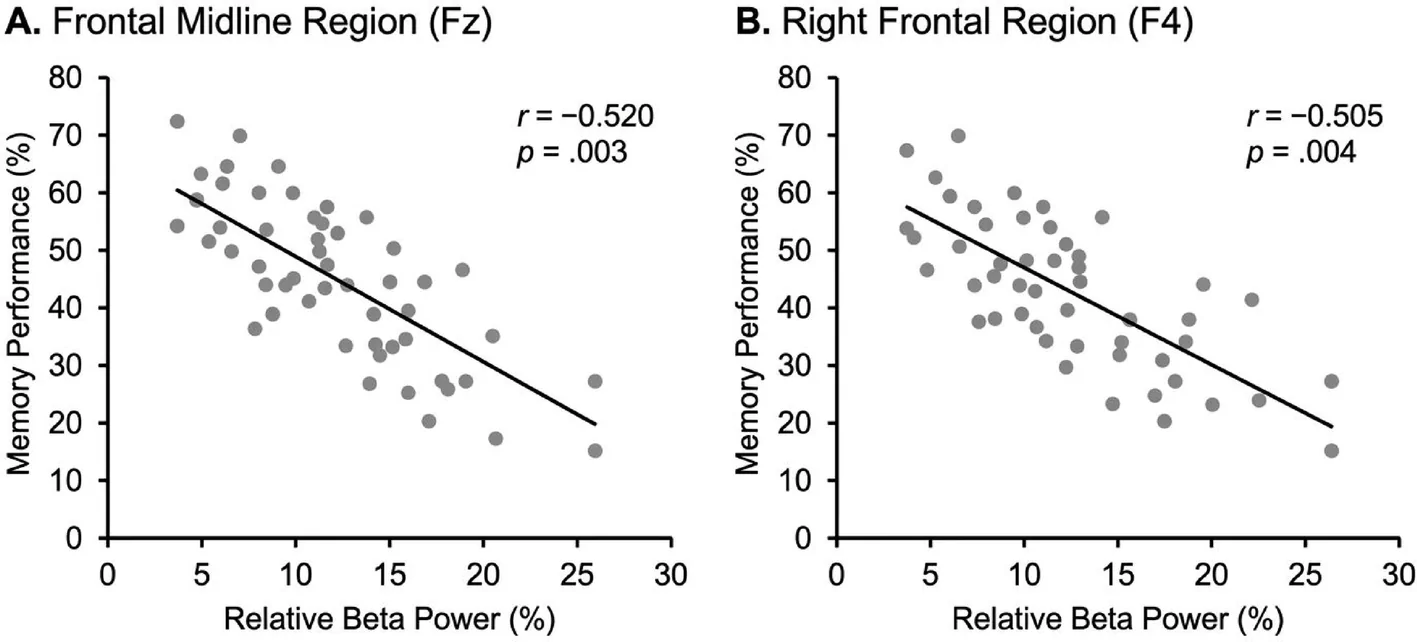
Scatterplots showing the relationships between relative beta power and memory performance in frontal regions. Panel **A** illustrates the significant negative correlation at the frontal midline region (Fz; r = -0.520, *p* = .003), and panel **B** shows the significant negative correlation at the right frontal region (F4; r = -0.505, *p* = .004). These results indicate that higher beta activity in these regions was associated with lower memory performance

**Fig. 4:**
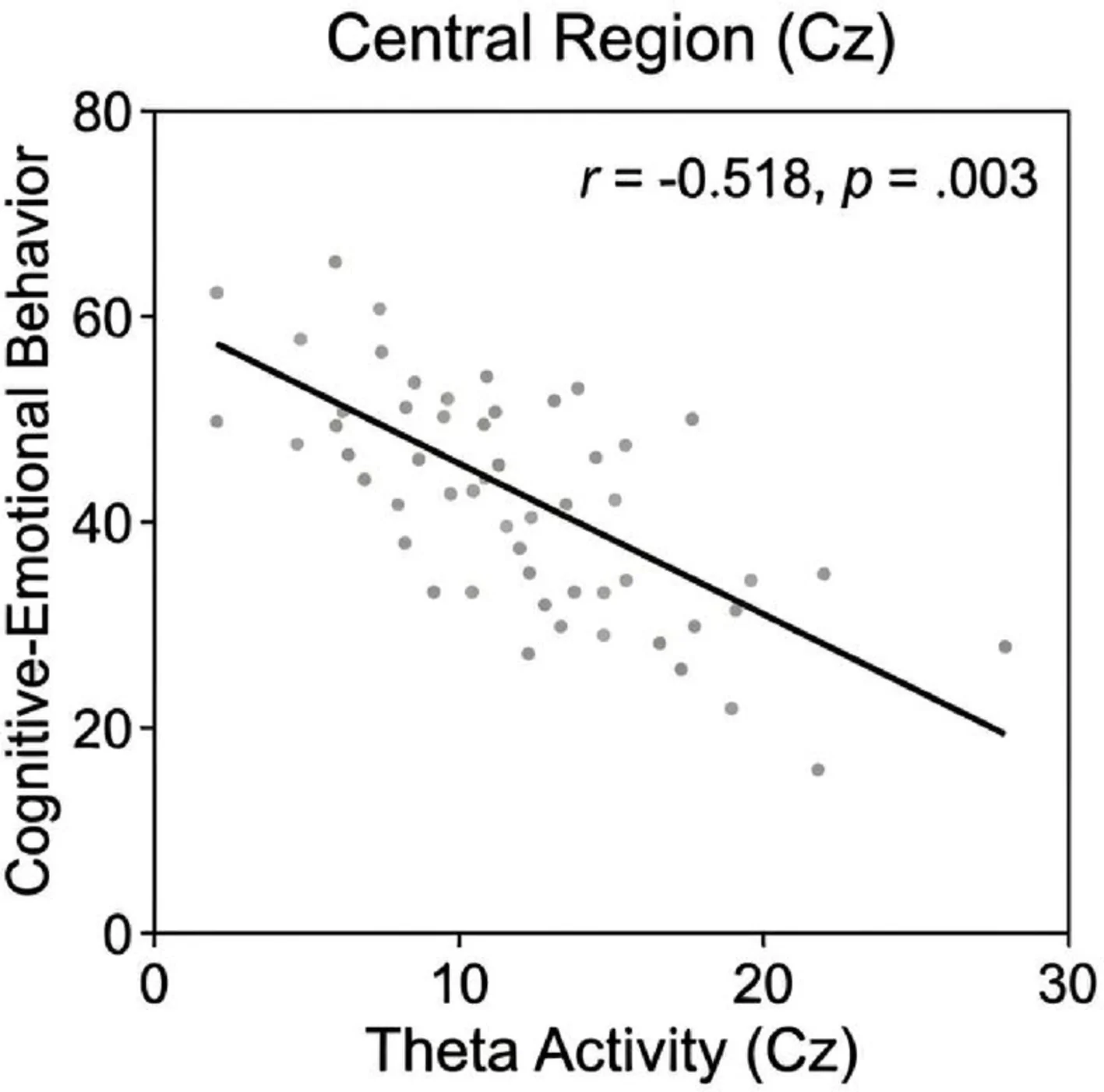
Scatterplot showing the relationship between theta activity during the eyes-closed condition and cognitive-emotional behavioral measures. A significant negative correlation was observed at Cz (r =  − 0.518, *p* = .003), indicating that higher theta activity was associated with lower scores on the cognitive-emotional behavioral measure

Negative correlations were observed between beta activity and memory performance across frontal regions (e.g., Fz: r =  − 0.520, *p* = 0.003; F4: r =  − 0.505, *p* = 0.004), indicating that higher beta activity was associated with lower memory performance (Fig. 3). Additionally, theta activity during eyes-closed conditions was negatively correlated with cognitive-emotional behavioral measures (e.g., Cz: r =  − 0.518, *p* = 0.003), suggesting a relationship between neural activity and cognitive-emotional regulation (Fig. 4).

## Discussion

This study examined task-related changes in QEEG theta relative power and theta/beta ratio across six scalp sites in individuals with schizophrenia under calm (eyes-closed), resting (eyes-open), and memory-task conditions. The findings suggest condition-dependent modulation of neural activity, particularly at frontal and central scalp sites, contributing to the understanding of cognitive and attentional processes in schizophrenia [20, 22].

### Frontal-midline theta and internal attention

The most consistent finding was that theta relative power was highest during the calm (eyes-closed) condition, particularly at frontal-midline (Fz) and central (Cz) sites. This pattern is consistent with previous studies linking frontal-midline theta to internally directed attention and cognitive control processes [6, 25]. However, elevated theta activity during the calm eyes-closed condition may also partly reflect reduced sensory input or vigilance-related factors that accompany eyes-closed recording. Therefore, the observed theta increase should be interpreted as reflecting both internally directed attention and reduced external sensory processing.

### Theta/beta ratio and cognitive state modulation

Theta/beta ratios showed a similar pattern, with the higher values during calm conditions and the lower values during memory task performance. This trend is consistent with previous findings suggesting that theta/beta ratio reflects changes in attentional engagement and cognitive state [11, 20]. However, as theta/beta ratio was analyzed descriptively in the present study, these findings should be interpreted cautiously. The reduction in ratio during memory tasks may reflect increased cognitive demand and associated beta activity, particularly in frontal and temporal regions.

### Regional differences in theta activity

Because the present findings are based on scalp-level QEEG recordings, regional interpretations should be considered cautious and should not be interpreted as precise cortical localization. The significant condition-by-electrode-site interaction indicates that theta modulation differed across scalp locations. Frontal and central sites (Fp1, Fz, Cz) consistently showed higher theta activity compared with the temporal site (T3), supporting their relevance to executive control and attentional processes [9, 13].

### Clinical implications

The findings have potential implications for cognitive and occupational therapy interventions in schizophrenia. The prominence of theta activity during calm, internally focused states may reflect neural readiness and cognitive regulation. Structured tasks that promote calm and focused attention, such as those used in the BMP protocol, may help inform targeted cognitive and occupational interventions aimed at improving attentional regulation and task engagement in individuals with schizophrenia [19]. These results support the integration of QEEG measures into occupation-based assessment and rehabilitation frameworks [4, 5]. Taken together, these findings suggest that internally directed calm states and externally driven task demands are associated with distinct patterns of scalp-level neural activity in schizophrenia.

### Limitations

Several limitations should be noted. First, the absence of a healthy control group limits the ability to determine whether the observed patterns are specific to schizophrenia or reflect more general cognitive processes. Second, the sample size was relatively modest, which may affect generalizability. Third, theta/beta ratio findings were descriptive and not supported by inferential statistical analyses. Fourth, individual alpha peak frequency (IAF) was not controlled, which may influence spectral measurements. Finally, no connectivity or source-level analyses were conducted; therefore, conclusions regarding network-level mechanisms or precise cortical localization remain preliminary.

## Conclusion

Theta activity at frontal and central scalp sites may reflect dynamic modulation between internally directed calm states and externally driven cognitive demands in schizophrenia. The highest theta activity observed during calm conditions may reflect internally directed attention and reduced sensory input, whereas reductions during memory tasks may reflect increased cognitive load.

## Data Availability

The datasets generated and/or analysed during the current study are not publicly available due to ethical and privacy restrictions but are available from the corresponding author on reasonable request.
